# Vaccine candidates for dengue virus type 1 (DEN1) generated by replacement of the structural genes of rDEN4 and rDEN4Δ30 with those of DEN1

**DOI:** 10.1186/1743-422X-4-23

**Published:** 2007-02-28

**Authors:** Joseph E Blaney, Neeraj S Sathe, Christopher T Hanson, Cai Yen Firestone, Brian R Murphy, Stephen S Whitehead

**Affiliations:** 1 Laboratory of Infectious Diseases, National Institute of Allergy and Infectious Diseases, National Institutes of Health, Bethesda, MD, 20892, USA

## Abstract

**Background:**

Antigenic chimeric viruses have previously been generated in which the structural genes of recombinant dengue virus type 4 (rDEN4) have been replaced with those derived from DEN2 or DEN3. Two vaccine candidates were identified, rDEN2/4Δ30(ME) and rDEN3/4Δ30(ME), which contain the membrane (M) precursor and envelope (E) genes of DEN2 and DEN3, respectively, and a 30 nucleotide deletion (Δ30) in the 3' untranslated region of the DEN4 backbone. Based on the promising preclinical phenotypes of these viruses and the safety and immunogenicity of rDEN2/4Δ30(ME) in humans, we now describe the generation of a panel of four antigenic chimeric DEN4 viruses using either the capsid (C), M, and E (CME) or ME structural genes of DEN1 Puerto Rico/94 strain.

**Results:**

Four antigenic chimeric viruses were generated and found to replicate efficiently in Vero cells: rDEN1/4(CME), rDEN1/4Δ30(CME), rDEN1/4(ME), and rDEN1/4Δ30(ME). With the exception of rDEN1/4(ME), each chimeric virus was significantly attenuated in a SCID-HuH-7 mouse xenograft model with a 25-fold or greater reduction in replication compared to wild type DEN1. In rhesus monkeys, only chimeric viruses with the Δ30 mutation appeared to be attenuated as measured by duration and magnitude of viremia. rDEN1/4Δ30(CME) appeared over-attenuated since it failed to induce detectable neutralizing antibody and did not confer protection from wild type DEN1 challenge. In contrast, rDEN1/4Δ30(ME) induced 66% seroconversion and protection from DEN1 challenge. Presence of the Δ30 mutation conferred a significant restriction in mosquito infectivity upon rDEN1/4Δ30(ME) which was shown to be non-infectious for *Aedes aegypti *fed an infectious bloodmeal.

**Conclusion:**

The attenuation phenotype in SCID-HuH-7 mice, rhesus monkeys, and mosquitoes and the protective immunity observed in rhesus monkeys suggest that rDEN1/4Δ30(ME) should be considered for evaluation in a clinical trial.

## Background

The dengue (DEN) viruses are members of the *Flaviviridae *family and contain a single-stranded positive-sense RNA genome [1]. A single viral polypeptide is cotranslationally processed by viral and cellular proteases generating three structural proteins (capsid C, membrane M, and envelope E) and at least seven non-structural (NS) proteins. The genome organization of the DEN viruses is 5'-UTR-C-prM-E-NS1-NS2A-NS2B-NS3-NS4A-NS4B-NS5-UTR-3' (UTR – untranslated region, prM – membrane precursor). Four dengue virus serotypes (DEN1, DEN2, DEN3, and DEN4) circulate in tropical and subtropical regions of the world inhabited by more than 2.5 billion people. There are an estimated 50 million dengue infections annually and hundreds of thousands of cases of dengue hemorrhagic fever (DHF), with children bearing much of the disease burden [2,3]. The increase in both the incidence and severity of disease caused by the four DEN serotypes over the past several decades has been well documented [4]. DEN viruses are maintained in a life cycle of transmission from mosquito to human to mosquito with no other apparent viral reservoir participating in this life cycle in urban settings [5].

An economical vaccine that prevents disease caused by the DEN viruses has become a global public health priority. The cost-effectiveness, safety, and long-term efficacy associated with the live attenuated vaccine against yellow fever (YF) virus, another mosquito-borne flavivirus, serves as a model for the feasibility of developing a live attenuated DEN virus vaccine [6]. We have employed two strategies for generating live attenuated vaccine candidates against each serotype which can then be combined into a tetravalent vaccine [7,8]. First, reverse genetics has been used to introduce an attenuating 30 nucleotide deletion (Δ30) mutation into the 3' untranslated region of cDNA clones of each DEN serotype [9-12]. In initial studies, the rDEN4Δ30 vaccine candidate was found to be attenuated in rhesus monkeys and phase I/II clinical trials in humans have demonstrated that virus infection results in low viremia, is strongly immunogenic, and exhibits minimal reactogenicity without serious adverse events [9,13]. The rDEN1Δ30 vaccine candidate, which was also attenuated in rhesus monkeys, has been found to share a similar set of properties in clinical trials as that observed for rDEN4Δ30; low viremia, strong immunogenicity, and minimal reactogenicity [14]. Importantly, both vaccines are highly immunogenic at a dose of 10^3 ^PFU/vaccinee indicating the feasibility for manufacture at low cost. Unfortunately, the rDEN2Δ30 and rDEN3Δ30 viruses were found to not be attenuated in rhesus monkeys [11,12]. Therefore, a second strategy for vaccine development was employed to develop the DEN2 and DEN3 components for the tetravalent DEN vaccine. This strategy involved the generation of antigenic chimeric viruses by replacement of the M and E structural proteins (ME) of the attenuated rDEN4Δ30 vaccine candidate with those from DEN2 or DEN3 yielding the rDEN2/4Δ30 and rDEN3/4Δ30 vaccine candidates, respectively [11,15]. During these studies it was found that antigenic chimerization of DEN2 or DEN3 with DEN4 yielded an attenuated virus. The rDEN2/4Δ30 vaccine virus has been tested in humans and appears safe, infectious, and strongly immunogenic at a dose of 10^3 ^PFU/vaccinee [16], while clinical evaluation of the rDEN3/4Δ30 virus is ongoing.

In this report, we extend the previous studies of rDEN2/4 and rDEN3/4 chimeric viruses by describing the generation of a panel of rDEN1/4 antigenic chimeric viruses using either the CME or ME structural genes of DEN1 Puerto Rico/94 on a DEN4 genetic background with and without the Δ30 mutation. The goal of this study was two-fold: to identify a suitable back-up vaccine candidate for the rDEN1Δ30 virus and to determine if DEN1, like DEN2 and DEN3, could be attenuated by chimerization with DEN4. Four antigenic chimeric viruses were generated and found to replicate efficiently in Vero cells: rDEN1/4(CME), rDEN1/4Δ30(CME), rDEN1/4(ME), and rDEN1/4Δ30(ME). The level of replication of these viruses was compared to that of wild type DEN1 or DEN4 in a SCID-HuH-7 mouse xenograft model, in rhesus monkeys, and in *Aedes aegypti *mosquitoes, and their immunogenicity and efficacy was evaluated in rhesus monkeys.

## Results

### Construction and recovery of chimeric DEN1 vaccine candidates

**Figure 1:**
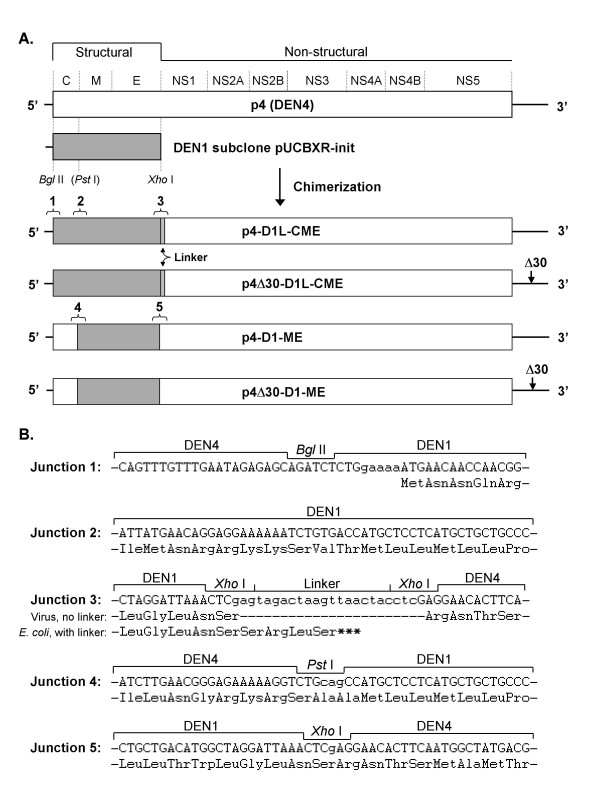
Molecular construction of the DEN1 chimeric cDNA plasmids. **A. **The CME structural protein coding region of the DEN4 cDNA plasmid p4 was replaced with the corresponding region from DEN1 (Puerto Rico/94) CME sub-clone pUCBXR-init. Following introduction of a *Pst*I site near the C-M junction of pUCBXR-init, the ME region of p4 was replaced with the corresponding region from DEN1. Restriction sites used to facilitate the construction are indicated. The virus genomic regions for each of the resulting full-length chimeric cDNA plasmids are shown and the presence of the Δ30 mutation is indicated in the 3'-UTR. DEN1 sequence is shaded. The resulting cloning junctions are numbered 1 – 5 and the inserted linker sequence is indicated as a hatched box. The location of the linker is indicated. **B. **The nucleotide sequences surrounding the cloning junctions identified above are shown along with the predicted amino acid sequence corresponding to the virus polyprotein. Lower case letters indicate nucleotide substitutions that differ from wild type. For junction 3, the amino acid sequence is shown as predicted for the virus genome (with the linker sequence removed prior to transcription) and as predicted in *E. coli *where cryptic expression of the virus polyprotein with the linker sequence intact would result in translational termination. The termination codon in the polyprotein open reading frame that is located in the linker is indicated by * * *. Termination codons exist in the linker sequence for each of the additional forward and reverse open reading frames. For junction 4, the introduced *Pst*I site is indicated. C = capsid protein; M = membrane protein (with precursor region), E = envelope protein; NS = nonstructural.

The chimerization of the structural genes of DEN1 Puerto Rico/94 with DEN4 was performed in a manner similar to methods used to construct the rDEN2/4 and rDEN3/4 chimeric viruses (Figure 1) [11,15]. Construction of a stable p4-D1-CME cDNA clone required the addition of a linker sequence between the E and NS1 genes as had been observed for the construction of the rDEN3/4 chimeric cDNA clones [11]. A total of four chimeric viruses were generated with either two (ME) or three (CME) DEN1 structural genes in the DEN4 background with or without the Δ30 mutation in the DEN4 3' UTR. These chimeric viruses are referred to as rDEN1/4(CME), rDEN1/4Δ30(CME), rDEN1/4(ME), and rDEN1/4Δ30(ME). Recombinant viruses were recovered in C6/36 cells and then were adapted to Vero cells by serial passage. Following adaptation, the viruses were biologically cloned by terminal dilution in Vero cells, and the complete nucleotide sequence of each chimeric virus was determined (Table 1). As expected, adventitious mutations were detected in the virus populations with each virus containing at least one amino acid change in NS4B, which is a previously identified locus for accumulation of mutations that enhance replication in Vero cells [17].

**Table 1:** Nonsynonymous adventitious mutations identified in rDEN1/4 chimeric viruses after passage in Vero cells

Virus	Gene/region	Nucleotide		Amino acid	
		
		Number^1^	Change	Number^2^	Change
rDEN1/4(CME)	NS4B	7134	A → G	102	Thr → Ala
	NS4B	7156	U → C	109	Val → Ala
rDEN1/4Δ30(CME)	NS2B	4269	A → G	45	Ser → Gly
	NS4B	7132	C → U	101	Pro → Leu
rDEN1/4(ME)	NS4B	7162	U → C	112	Leu → Ser
rDEN1/4Δ30(ME)	prM	603	C → G	55	Arg → Gly
	NS4B	7182	G → C	119	Gly → Arg
	3' UTR	10471	G insertion	--	--

^1 ^Nucleotide numbering is from 5' genome end.

^2 ^Amino acid numbering is according to position in respective protein product.

**Figure 2:**
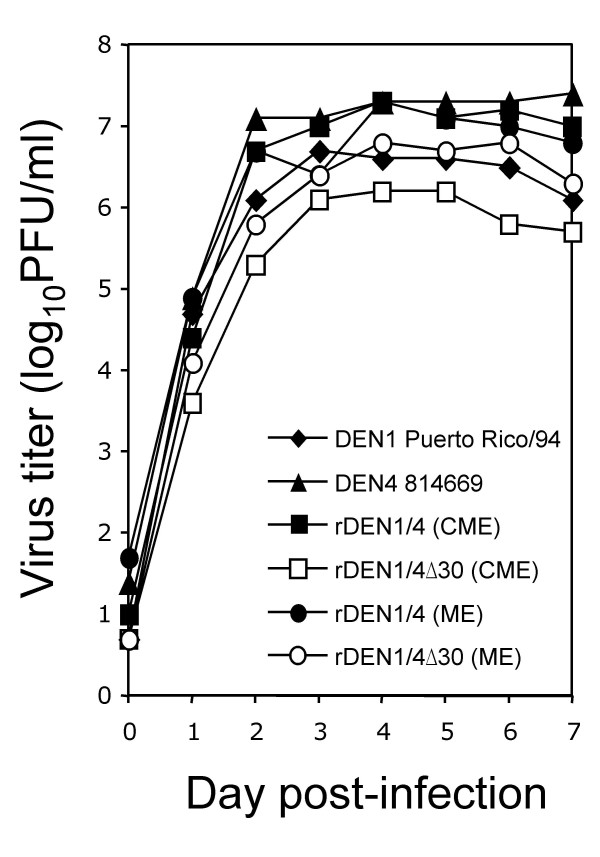
Virus replication in Vero cells. Virus titer in supernatants from infected cells was determined by plaque assay in Vero cells. The lower limit of detection was 0.7 log_10 _PFU/ml.

Chimeric viruses were assessed for replication in Vero cells, which are the intended cell substrate for vaccine manufacture (Figure 2). At a multiplicity of infection of 0.01, rDEN1/4(CME) and rDEN1/4(ME) each reached a virus titer of 7.3 log_10 _PFU/ml, which approximates the yield of wild type DEN4 infection. Inclusion of the Δ30 mutation resulted in marginally reduced replication, but even rDEN1/4Δ30(CME), which had the lowest level of replication, reached a virus titer of 6.2 log_10 _PFU/ml. Thus, after adaptation to growth in Vero cells, each chimeric virus replicates to a level that would allow for efficient manufacture of a vaccine virus.

### Replication of chimeric viruses in SCID-HuH-7 mice

The level of replication of rDEN1/4 chimeric viruses was first evaluated in SCID-HuH-7 mice since the level of replication in these mice is expected to correlate with the level of attenuation in rhesus monkeys [7]. In this SCID mouse model, tumors which develop from implanted human hepatoma cells are infected with DEN viruses, and viremia is assessed at seven days post-infection. Wild type DEN4 has previously been shown to replicate to 6.0 log_10 _PFU/ml in SCID-HuH-7 mice [18]. Wild type DEN1 Puerto Rico/94 replicated to a mean peak virus titer of 6.6 log_10 _PFU/ml (Table 2), which is similar to replication levels observed for the other wild type DEN viruses [11,12]. Replication of both CME chimeric viruses, rDEN1/4(CME) and rDEN1/4Δ30(CME), was significantly restricted when compared to the wild type DEN1 level of replication with a 40-fold and 159-fold reduction in replication, respectively. The addition of the Δ30 mutation did not appear to confer significant additional attenuation upon the rDEN1/4(CME) chimeric virus.

**Table 2:** rDEN1/4 chimeric virus replication in SCID-HuH-7 mice.

Virus^1^	No. mice	Peak virus titer (log_10 _PFU/ml ± SE)	Fold reduction from wild type DEN1
DEN1 Puerto Rico/94^2^	18	6.6 ± 0.1	--
rDEN1/4(CME)	8	5.0 ± 0.4^3^	40
rDEN1/4Δ30(CME)	7	4.4 ± 0.4^3^	159
rDEN1/4(ME)	9	6.8 ± 0.3	-2
rDEN1/4Δ30(ME)	7	5.2 ± 0.5^3^	25

^1 ^Groups of SCID-HuH-7 mice were inoculated into the peritoneal tumor with 4.0 log_10 _PFU of the indicated virus. Serum was collected on day 7, and virus titer was determined in Vero cells.

^2 ^Data combined from 2 experiments.

^3 ^Significant reduction compared to wild type DEN1 (Tukey post-hoc test, *P *< 0.05).

In contrast, the rDEN1/4(ME) virus reached a peak virus titer of 6.8 log_10 _PFU/ml and therefore, was not attenuated for SCID-HuH-7 mice. rDEN1/4Δ30(ME) was significantly restricted in replication compared to wild type DEN1 with a 25-fold reduction in replication indicating that the Δ30 mutation was attenuating when added to the rDEN1/4(ME) virus. These results indicate that both the Δ30 mutation and the virus origin of the C gene play a role in the attenuation of rDEN1/4 viruses in SCID-HuH-7 mice.

### Replication of chimeric viruses in rhesus monkeys

Wild type DEN1 Puerto Rico/94 was found to replicate efficiently in rhesus monkeys comparable to previous studies with the DEN1 Western Pacific strain [10]. All of the animals infected with DEN1 Puerto Rico/94 developed viremia with a mean duration of 2.8 days and mean peak virus titer of 2.0 log_10 _PFU/ml (Table 3). The rDEN1/4(CME) virus appeared to be only marginally attenuated, if at all, when compared to DEN1 Puerto Rico/94 in terms of mean number of viremic days and mean peak virus titer. However, rDEN1/4Δ30(CME) was found to be attenuated when compared to wild type DEN1 virus and the parent rDEN1/4(CME) virus. Viremia was detected in only half of monkeys infected with rDEN1/4Δ30(CME), and both the mean duration (0.5 days) and mean peak virus titer (1.1 log_10 _PFU/ml) were less than that of both wild type DEN1 and rDEN1/4(CME).

**Table 3:** Replication and immunogenicity of rDEN1/4 chimeric viruses in rhesus monkeys.

Virus^1^	No. of monkeys	% of monkeys with viremia	Mean no. of viremic days per monkey	Mean peak virus titer^2 ^(log_10 _PFU/ml ± SE)	Geometric mean serum neutralizing antibody titer (reciprocal dilution)^3^			Post-challenge^5^	
						
					Day 0	Day 28	% sero-conversion^4^	% of monkeys with viremia	Mean peak virus titer^2 ^(log_10 _PFU/ml ± SE)
DEN1 Puerto Rico/94	4	100	2.8	2.0 ± 0.1	< 5	103	100	0	< 1.0
rDEN1/4 (CME)	4	100	2.0	1.5 ± 0.1	< 5	56	100	0	< 1.0
rDEN1/4Δ30 (CME)	4	50	0.5	1.1 ± 0.1	< 5	< 5	0	75	1.7 ± 0.3
rDEN1/4 (ME)	4	100	4.0	2.1 ± 0.1	< 5	65	100	0	< 1.0
rDEN1/4Δ30 (ME)	6	50	0.7	1.1 ± 0.2	< 5	23	66	0	< 1.0
Mock infected	2	0	0	< 1.0	< 5	< 5	0	100	2.1 ± 0.2

^1 ^Groups of rhesus monkeys were inoculated subcutaneously on day 0 with 5.0 log_10 _PFU of the indicated virus in a 1 ml dose. Serum was collected daily for 10 days to detect viremia and on day 28 for determination of neutralizing antibodies.

^2 ^Virus titer in serum was determined by plaque assay in Vero cells.

^3 ^Plaque reduction (60%) neutralizing antibody titers were determined using DEN1 Puerto Rico/94 as test virus.

^4 ^Percentage of monkeys with a four-fold or greater rise in neutralizing antibody levels between day 0 and 28.

^5 ^Monkeys were challenged after 35 days with DEN1 (Puerto Rico/94) administered subcutaneously in a 1 ml dose containing 5.0 log_10 _PFU. Serum was collected daily for 10 days to detect viremia.

As had been observed in SCID-HuH-7 mice, infection of rhesus monkeys with rDEN1/4(ME) yielded no evidence of attenuation and the virus replicated to levels similar to that of wild type DEN1. In contrast, replication of rDEN1/4Δ30(ME) was found to be restricted when compared to either wild type DEN1 or rDEN1/4(ME) and was similar to that of rDEN1/4Δ30(CME). Fifty percent of monkeys infected with rDEN1/4Δ30(ME) developed detectable viremia. There was a four-fold reduction in the mean duration of viremia (0.7 days) and nearly a ten-fold reduction in mean peak virus titer (1.1 log_10 _PFU/ml) compared to wild type DEN1.

The level of serum neutralizing antibody titer was determined in monkeys 28 days post-immunization (Table 3). Infection with wild type DEN1, rDEN1/4(CME), or rDEN1/4(ME) resulted in 100% seroconversion, defined as a four-fold or greater rise in serum neutralizing antibody levels after immunization. Neutralizing antibodies were not detected in any of the four monkeys immunized with rDEN1/4Δ30(CME) despite the presence of a low level of viremia in two monkeys, suggesting that this DEN1/4 chimeric virus may be over-attenuated. In contrast, four of six monkeys immunized with rDEN1/4Δ30(ME) seroconverted with a geometric mean serum neutralizing antibody titer of 1:23 which was approximately four-fold reduced from wild type DEN1 Puerto Rico/94 (1:103).

All immunized monkeys were challenged with wild type DEN1 virus on day 35 post-immunization and resulting viremia was quantified (Table 3). Both mock-immunized monkeys developed viremia after challenge with a mean peak virus titer of 2.1 log_10 _PFU/ml. Prior immunization with rDEN1/4(CME) induced complete protection from detectable viremia after challenge. rDEN1/4Δ30(CME), which was poorly immunogenic, failed to confer protection in three of four challenged monkeys. This result confirms the over-attenuation of the rDEN1/4Δ30(CME) virus in rhesus monkeys. Both of the (ME) chimeric viruses, rDEN1/4(ME) or rDEN1/4Δ30(ME), conferred complete protection from viremia following wild type DEN1 challenge including those animals that did not develop detectable levels of serum antibody.

### Replication of (ME) chimeric viruses in mosquitoes

Based on the high level of attenuation of rDEN1/4Δ30(ME) virus in SCID-HuH-7 mice and in rhesus monkeys and on its protective efficacy in rhesus monkeys, it appeared that this chimeric virus might be suitable for further study in humans. Therefore, it was tested for infectivity and replication in *Aedes aegypti *mosquitoes to assess its potential for transmissibility. The infectivity and level of replication of rDEN1/4Δ30(ME) chimeric virus was compared to those of wild type parental DEN1 and DEN4 and of rDEN1/4(ME) following oral feeding of virus in a bloodmeal. Specifically, following ingestion of an infectious bloodmeal that contained 10^4.3 ^PFU/2 ul, virus replication in the midgut and dissemination to the salivary glands was determined by plaque titration of virus in the mosquito body and head, respectively. Wild type DEN1 and DEN4 replicated efficiently in the midgut of mosquitoes and dissemination to the salivary glands was robust with 88% and 71% of DEN1-infected or DEN4-infected mosquitoes experiencing disseminated infections, respectively (Table 4). 100% of mosquitoes with DEN1 midgut infections developed disseminated infections, while 81% of mosquitoes with DEN4 midgut infections had disseminated infections. DEN1 reached mean virus titers that were approximately 10-fold to 100-fold higher than DEN4 in both the body and head. rDEN1/4(ME) virus had slightly lower infectivity for the midgut, but dissemination to the head was comparable to that of its DEN4 wild type parent. The mean virus titer in the bodies (2.4 log_10 _PFU/body) or heads (3.0 log_10 _PFU/head) of rDEN1/4(ME)-infected mosquitoes approximated the values observed for wild type DEN4. However, presence of the Δ30 mutation in rDEN1/4Δ30(ME) conferred a profound reduction in mosquito infectivity. Virus infection was not detected in the bodies (or heads) of the 24 mosquitoes that were fed an infectious bloodmeal containing rDEN1/4Δ30(ME) suggesting that the rDEN1/4Δ30(ME) chimeric virus would be poorly transmissible by mosquitoes.

**Table 4:** Replication of rDEN1/4(ME) chimeric viruses in *Aedes aegypti*

Virus^1^	No. mosquitoes	Body^2^		Head^2^		% Dissemination^3^
				
		% infected	Mean virus titer (log_10 _PFU/body)	% infected	Mean virus titer (log_10 _PFU/head)	
DEN1	24	88	4.5 ± 0.1	88	4.7 ± 0.1	100
DEN4	24	88	3.0 ± 0.2	71	2.7 ± 0.3	81
rDEN1/4(ME)	24	63	2.4 ± 0.3	42	3.0 ± 0.4	67
rDEN1/4Δ30(ME)	24	0	< 0.4	0	< 0.4	0

^1 ^Assuming a 2 ul bloodmeal, 4.3 log_10 _PFU virus of each virus were ingested.

^2 ^Presence of virus in mosquito homogenates was determined by plaque assay in Vero cells. Mean virus titer was determined using values only from samples with detectable virus.

^3 ^Calculated as no. infected heads/no. infected bodies.

## Discussion

Since the first report of the generation of an antigenic chimeric dengue virus using reverse genetics [19], numerous viruses have been created using genes from tick-borne and mosquito-borne flaviviruses, and many of these viruses have potential usefulness as live attenuated virus vaccines [15,20-25]. Recently, clinical studies have indicated that antigenic chimeric flaviviruses may in fact be a safe and an effective means of vaccination for protection against disease caused by the DEN viruses, Japanese encephalitis virus (JEV), and West Nile virus (WNV) [16,26-28]. Specifically, the rDEN2/4Δ30(ME) vaccine candidate was found to be safe in humans and induced a potent neutralizing antibody response against DEN2 [16]. In addition, using the yellow fever virus 17D vaccine as the genetic background, antigenic chimeric viruses expressing the M and E genes of DEN2 [26], JEV [28], and WNV [27], have been tested in humans and found to be safe and strongly immunogenic. These promising clinical studies indicate that continued development of antigenic chimeric flaviviruses as live attenuated virus vaccines is a rational and important pursuit. In the present study, an additional antigenic chimeric virus, rDEN1/4Δ30(ME), is identified that has an attenuation and immunogenicity phenotype similar to the previously evaluated rDEN2/4Δ30(ME) and rDEN3/4Δ30(ME) vaccine candidates [11,15]. Studies were designed to assess the relative contribution of (1) chimerization, (2) the parental origin of the C gene in the DEN1/4 chimeric virus, and (3) the Δ30 mutation to the attenuation of the DEN1/4 chimeric viruses for SCID-HuH-7 mice, rhesus monkeys, and mosquitoes.

In previous studies, chimeric flaviviruses generated by substitution of the ME proteins of a wild type flavivirus such as WNV, Langat, DEN2 or DEN3, for the ME genes of the DEN4 wild type virus, were uniformly attenuated for rodents or rhesus monkeys [11,15,20,22]. This indicated that chimerization resulted in attenuation of each chimeric flavivirus that was evaluated. Based on these observations, we fully expected that the rDEN1/4(ME) chimeric virus generated in the present study would be attenuated in SCID-HuH-7 mice or rhesus monkeys. However, this was not observed. The rDEN1/4(ME) chimeric virus replicated like parental wild type virus in SCID-HuH-7 mice, rhesus monkeys, and mosquitoes. The ability of chimerization to attenuate rDEN2/4(ME) and rDEN3/4(ME), but not rDEN1/4(ME), cannot be explained by differences in the genetic relatedness among these viruses. The amino acid homology of the DEN1, DEN2, and DEN3 structural genes with those of DEN4 ranges from 63% to 67%. Since the factors that control attenuation resulting from chimerization have not been clearly identified, it would appear that each antigenic chimeric virus that is generated will need individual characterization to define the genetic factors contributing to its observed attenuation. In the case of rDEN1/4(ME), chimerization does not result in attenuation, and therefore attenuation of rDEN1/4(ME) chimeric viruses must rely on genetic factors other than chimerization itself.

The contribution of the parental origin of the C protein in the DEN1/4 chimeric viruses to attenuation was assessed by comparing the level of attenuation of rDEN1/4(ME) and rDEN1/4(CME) in SCID-HuH-7 mice and rhesus monkeys. Since rDEN1/4(ME) was not attenuated in these animals, it was possible to determine whether the parental origin of the C gene could influence the level of replication in these hosts. It was previously found that both rDEN2/4(CME) and rDEN2/4(ME) viruses resulted in a similar level of attenuation in SCID-HuH-7 mice, rhesus monkeys and mosquitoes indicating that the parental origin of the C protein in the rDEN2/4 chimeric viruses did not modify the level of attenuation resulting from chimerization [15]. Thus, a contribution of the C protein to attenuation was not identified in the DEN2/4 chimeric viruses. In the present study, rDEN1/4(CME), but not rDEN1/4(ME) was significantly attenuated in SCID-HuH-7 mice, but not in rhesus monkeys. In addition rDEN1/4Δ30(CME) was more attenuated than rDEN1/4Δ30(ME) in SCID-HuH-7 mice and rhesus monkeys. These findings indicate that the presence of the DEN1 C protein in the rDEN1/4(CME) and rDEN1/4Δ30(CME) had an independent attenuating effect. Thus, the presence of a DEN1 C protein in a DEN4 backbone resulted in restricted replication, which was somewhat dependent upon the particular animal model and the presence or absence of the Δ30 mutation. It is not surprising that the presence of a heterologous C gene might attenuate a chimeric DEN virus as the C gene is known to be involved in packaging of the RNA genome. The C gene has been shown to interact specifically with DEN virus RNA [29]. It is possible that this interaction may be governed by serotype-specific interactions which could explain why the CME chimeric virus might be more attenuated than the ME chimeric virus.

Presence of the Δ30 deletion mutation in rDEN1/4Δ30(ME) or rDEN1/4Δ30(CME) resulted in a restriction of replication of the chimeric viruses for SCID-HuH-7 mice and rhesus monkeys indicating that this mutation had a strong attenuating effect for each chimeric virus. In the case of rDEN1/4Δ30(CME), the presence of the Δ30 mutation resulted in over-attenuation in rhesus monkeys. This virus clearly had two factors contributing to attenuation, namely, the Δ30 deletion mutation and the presence of the DEN1 C gene. The over-attenuation of rDEN1/4Δ30(CME) parallels what was observed for the rDEN2/4Δ30(CME) virus where the presence of the Δ30 mutation yielded a virus that did not infect rhesus monkeys, and like the rDEN1/4Δ30(CME) virus, was over-attenuated for rhesus monkeys [15]. The phenotype of rDEN1/4Δ30(ME) indicated a strong effect of the Δ30 mutation on attenuation since the rDEN1/4(ME) virus had no discernible level of attenuation. This is in contrast to the studies of rDEN2/4Δ30(ME) and rDEN3/4Δ30(ME) where inclusion of the Δ30 mutation did not appear to augment the level of attenuation attributable to chimerization [11,15]. For these viruses, chimerization itself provided the dominant attenuating effect which may have functionally masked the attenuation phenotype provided by the Δ30 mutation.

The rDEN1/4Δ30(ME) virus has many properties that make it an attractive vaccine candidate, and most of these properties are shared by rDEN2/4Δ30(ME) and rDEN3/4Δ30(ME) which are presently in clinical evaluation. rDEN1/4Δ30(ME) was attenuated in SCID-HuH-7 mice with a 25-fold reduction in replication compared to the parental wild type DEN virus, and rDEN2/4Δ30(ME) and rDEN3/4Δ30(ME) were similarly restricted in this host [11,15]. In rhesus monkeys, infection with rDEN1/4Δ30(ME) resulted in detectable viremia in 50% of monkeys, whereas 25% and 0% of rhesus monkeys infected with rDEN2/4Δ30(ME) or rDEN3/4Δ30(ME) had viremia, respectively [11,15]. Immunization with rDEN1/4Δ30(ME) resulted in a mean number of viremic days of less than one and a reduced mean peak virus titer compared to the wild type parent virus, and these findings were similar to those found with rDEN2/4Δ30(ME). Immunization with rDEN1/4Δ30(ME), rDEN2/4Δ30(ME), or rDEN3/4Δ30(ME) protected rhesus monkeys from challenge. Finally, replication of the chimeric viruses was attenuated for *Ae. aegypti *mosquitoes indicating that each of the three antigenic chimeric viruses would likely manifest decreased transmissibility by mosquitoes [11,15]. These results indicate remarkably similar phenotypes for these three viruses, suggesting that the three antigenic chimeric viruses could be combined with rDEN4Δ30 to form a tetravalent formulation that may exhibit balanced replication and immunogenicity. For a tetravalent vaccine formulation, achieving a balance of infectivity among the components is essential and has proven in the past to be a difficult goal to achieve [30,31].

Where does the rDEN1/4Δ30(ME) virus fit into our overall plans to develop a tetravalent DEN virus vaccine? At present, the rDEN1Δ30 virus, which contains the Δ30 mutation in the context of the full-length DEN1 genome, appears to be our most promising DEN1 vaccine candidate for inclusion in a tetravalent formulation [10]. The rDEN1Δ30 virus was found to be significantly attenuated in SCID-HuH-7 mice and rhesus monkeys when tested as a monovalent vaccine, and it was found to be an immunogenic and protective component of a tetravalent vaccine formulation in rhesus monkeys [8,10]. In addition, a recent phase I clinical trial in 20 volunteers indicated that rDEN1Δ30 was safe and strongly immunogenic [14]. However, should the rDEN1Δ30 virus prove not to be a suitable component of a tetravalent formulation when tested in humans, the rDEN1/4Δ30(ME) vaccine candidate, described here, would be a reasonable back-up candidate based on its level of attenuation in SCID-HuH-7 mice, rhesus monkeys, and mosquitoes and its immunogenicity and protective efficacy in monkeys.

## Conclusion

The construction of rDEN1/4 chimeric viruses with either DEN1 CME or ME regions in the presence or absence of the Δ30 mutation resulted in viruses with varying degrees of attenuation in animal models and mosquitoes. These results and our previous studies of chimeric DEN2/4 and DEN3/4 viruses indicate that the degree of attenuation conferred by the chimerization of flaviviruses can be influenced by three factors: the serotype of the donor structural genes, the constellation of the structural genes (CME or ME), and the contribution of additional mutations such as Δ30. Of the four DEN1 chimeric viruses evaluated in this study, rDEN1/4Δ30(ME) appears to have the most promising preclinical phenotype, characterized by attenuation in SCID-HuH-7 mice, rhesus monkeys, and mosquitoes. The rDEN1/4Δ30(ME) vaccine candidate appears to be suitable for evaluation in a clinical trial.

## Methods

### Cells and viruses

Vero cells (African green monkey kidney) were grown in OptiPro SFM (Invitrogen, Grand Island, NY) supplemented with 4 mM L-glutamine (Invitrogen). HuH-7 cells (human hepatoma) were maintained in D-MEM/F-12 (Invitrogen) supplemented with 10% fetal bovine serum (FBS), 1 mM L-glutamine and 0.05 mg/ml gentamicin (Invitrogen). C6/36 cells (*Aedes albopictus *mosquito) were maintained at 32°C in Minimal Essential Medium (MEM) containing Earle's salts and 25 mM HEPES buffer (Invitrogen) and were supplemented with 10% FBS, 2 mM L-glutamine, and 0.1 mM non-essential amino acids (Invitrogen). The DEN1 Puerto Rico/94 virus was provided by Dr. Duane Gubler (John A. Burns School of Medicine, University of Hawaii).

### Genetic construction of chimeric DEN1 viruses

Chimeric viruses have been generated in which the CME and ME genes of the DEN4 p4 and p4Δ30 cDNA clones have been replaced with the corresponding genes derived from DEN1 Puerto Rico/94 (Figure 1). The genomic region within nucleotides 72 – 2353 was amplified by RT-PCR from the DEN1 Puerto Rico/94 genome using DNA primers to introduce a single nucleotide change at 2344 to create a translationally-silent *Xho*I restriction enzyme recognition site (Figure 1B). The resulting PCR fragment was cloned blunt-ended into vector pCR2.1 (Invitrogen) from which a 2255 bp fragment was obtained following partial digestion with *Bgl*II (DEN1 nucleotide 86) and *Xho*I (DEN1 nucleotide 2341). This 2255 bp fragment was cloned into a modified pUC118 vector containing the *Asc*I – *Bgl*II fragment (SP6 promoter and DEN4 5'-UTR) from plasmid p4 (GenBank Accession number, AY648301). To generate a 5' untranslated region that would be identical to that of DEN4, site-directed mutagenesis was used to insert the nucleotides GAAAA immediately upstream of the AUG initiation codon for the polyprotein (Figure 1B, Junction 1), and the resulting plasmid was designated as pUCBXR-init.

To generate full-length CME chimeric cDNA plasmids, the *Asc*I – *Xho*I region of p4 or p4Δ30 was replaced with the corresponding fragment derived from DEN1 subclone pUCBXR-init. Initially, ligation of the *Asc*I – *Xho*I fragment of pUCBXR-init into p4 or p4Δ30 failed to yield genetically stable full-length cDNA plasmids. However, ligation in the presence of a synthetic DNA linker molecule containing termination codons in each of the forward and reverse coding frames and flanked by *Xho*I overhangs yielded stable plasmids p4-D1L-CME and p4Δ30-D1L-CME. The nucleotide and amino acid sequences of the resulting junctions are shown in Figure 1B.

To generate ME chimeric cDNA plasmids, site-directed mutagenesis of pUCBXR-init was used to introduce a unique *Pst*I restriction enzyme recognition site at DEN1 nucleotide 400 by substituting nucleotides 399–401 (UGA → CAG) immediately downstream of coding sequence for the trypsin cleavage site separating the C-protein from its anchor region. Introduction of the *Pst*I cleavage site is translationally silent for the DEN4 portion of the chimeric molecule; however, DEN1 C-protein amino acids 102 – 103 are changed from Val – Thr to Ala – Ala in the chimeric molecule (Figure 1B). A 1939 bp *Pst*I – *Xho*I fragment derived by partial digestion of the resulting plasmid was cloned into the *Pst*I – *Xho*I window of a pUC119 clone containing the *Asc*I – *Xho*I region of DEN4 clone p4 to create plasmid pUC-D1(ME). To generate full-length ME chimeric cDNA plasmids, the *Asc*I – *Xho*I region of p4 or p4Δ30 was replaced with the corresponding fragment derived from DEN1 subclone pUC-D1(ME). The resulting cDNA plasmids, p4-D1-ME and p4Δ30-D1-ME, were genetically stable in *E. coli *and did not require the *Xho*I synthetic linker fragment. The genomic region of each chimeric cDNA was sequenced as previously described [9] and GenBank accessions were assigned as follows (plasmid: accession numbers): p4-D1L-CME: EF456758, p4Δ30-D1L-CME: EF456759, p4-D1-ME: EF456756, p4Δ30-D1-ME: EF456757.

### Recovery and propagation of rDEN1 viruses

Plasmids were linearized with *Acc*65I (isoschizomer of *Kpn*I which cleaves leaving only a single 3' nucleotide), and transcribed in vitro using SP6 polymerase. Prior to linearization, the *Xho*I linker was excised from p4-D1L-CME and p4Δ30-D1L-CME to restore the intact coding sequence. Briefly, the p4-D1L-CME and p4Δ30-D1L-CME plasmids were digested with *Xho*I and purified to remove the short linker. The plasmids were then circularized by DNA ligase and linearized by *Acc*65I.

Purified transcripts were transfected into Vero or C6/36 cells using DOTAP liposomes (Roche, Indianapolis, IN). After recovery, viruses were passaged in Vero cells until virus titers reached approximately 10^6 ^PFU/ml. Viruses were subsequently biologically cloned by two or three terminal dilutions before experimental stocks were grown in Vero cells. The nucleotide sequence of each recovered virus was determined as previously described [9].

For analysis of replication in tissue culture, confluent 75 cm^2 ^flasks of Vero cells were infected with virus at a multiplicity of infection of 0.01. Aliquots of 0.5 ml were removed from flasks daily for seven days. After addition of SPG stabilizer (final concentration: 218 mM sucrose, 6 mM L-glutamic acid, 3.8 mM monobasic potassium phosphate, and 7.2 mM dibasic potassium phosphate, pH 7.2), samples were frozen on dry ice and stored at -80°C. Virus titer was determined by plaque assay on Vero cells. The limit of detection was 0.7 log_10 _PFU/ml.

### Animal models of DEN virus infection

For analysis of virus replication in SCID-HuH-7 mice, four to six week-old SCID mice (Tac:Icr:Ha(ICR)-Prkdc^scid^) (Taconic, Germantown, NY) were injected intraperitoneally with 0.1 mL phosphate-buffered saline containing 10^7 ^HuH-7 cells [32]. Five to six weeks after transplantation, tumor-bearing mice were infected by direct inoculation into the tumor with 10^4 ^PFU of virus in 50 μl Opti-MEM I (Invitrogen). Serum was collected from infected mice on day 7 post-infection and frozen at -80°C. Virus titer was determined by plaque assay in Vero cells [9].

DEN1 viruses were evaluated for replication and immunogenicity in rhesus macaques using previously described methods [9]. Dengue virus seronegative monkeys were inoculated subcutaneously with 10^5 ^PFU of virus diluted in L-15 medium (Invitrogen) or with a mock inoculum of L-15 medium. Serum was collected on days 0–6, 8, 10 and 28 after inoculation and stored at -80°C. Virus titer in serum was determined for each day by plaque assay in Vero cells and serum neutralizing antibody titer was determined for days 0 and 28 by plaque reduction neutralization test in Vero cells [9]. On day 35 post-infection, all monkeys were challenged by subcutaneous infection with 10^5 ^PFU of DEN1 wild type virus. Serum was collected on days 0–8 and on day 10 for virus titration and frozen at -80°C.

### Replication in mosquitoes

Virus replication was evaluated in *Aedes aegypti *provided by Nancy McLean-Cooper (Walter Reed Army Institute for Research). Mosquitoes were fed an infectious bloodmeal and incubated for 21 days as previously described [33]. Assuming a 2 ul bloodmeal, 4.3 log_10 _PFU of virus was ingested by the mosquitoes. After freezing, heads and bodies were dissected and homogenized in 250 ul suspension buffer (Hank's balanced salt solution). Virus titer in homogenates was determined by plaque assay in Vero cells.

## Competing interests

The vaccine candidates described here have been patented by the National Institute of Allergy and Infectious Diseases (NIAID). Through the execution of licensing agreements, the NIAID makes the vaccine candidates available to parties interested in their further development and commercialization.

## Authors' contributions

JEB recovered viruses, performed animal studies and drafted the manuscript. NSS performed growth curves and participated in animal studies. CTH conducted sequencing and mosquito studies. CYF participated in plasmid construction. SSW recovered viruses and BRM and SSW supervised the study and participated in its design and planning. All authors read and approved the final manuscript.
